# Acute Chest Pain and Broad Complex Tachycardia. A Non-typical Case of Pre-excited Atrial Fibrillation

**DOI:** 10.4021/cr70e

**Published:** 2011-07-25

**Authors:** Ramon Suarez Arias, Nuria Perez Villanueva, Gustavo Iglesias Cubero, Jose Rubin Lopez

**Affiliations:** a Cardiology Unit, Alvarez Buylla Hospital, Mieres (Asturias), Spain; b Emergency Department, Alvarez Buylla Hospital, Mieres (Asturias), Spain; c Cardiology Department, Hospital Universitario Central de Asturias, Oviedo, Spain; d Cardiology Department - Arrhythmia Unit, Hospital Universitario Central de Asturias, Oviedo, Spain

**Keywords:** Broad complex tachycardia, Pre-excited atrial fibrillation, Wolff-Parkinson-White syndrome

## Abstract

Wolff-Parkinson-White syndrome is a common condition in the emergency department. A case is presented of a 76-year-old patient with acute chest pain and broad complex tachycardia. Despite the fact that previous and post cardioversion ECG tracings in sinus rhythm showed no signs of pre-excitation, the characteristic pattern of pre-excited atrial fibrillation (AF) is recognized and after successful DC cardioversion the patient is referred for catheter ablation of the accessory pathway. This case illustrates a non-typical presentation of the WPW syndrome, with an older patient than usual with slight signs of pre-excitation. We highlight the need for high grades of suspicion for the early recognition of pre-excited AF when attending patients with tachycardia and the obligation to know the distinctive aspects of its management for this potentially life-threatening arrhythmia.

## Introduction

In foetal life, the atria and ventricles are eventually separated by a fibrous plate called the atrioventricular (AV) ring. The AV ring provides support for the mitral and tricuspide valves and electrically insulates the atria and ventricles. Normally the atria become isolated from the ventricles during foetal development, apart from the AV junction (i.e. AV node plus His bundle). The AV node is the only structure that should allow conduction through the AV ring. After birth, if there is an incomplete separation, residual muscle fibres may bridge the AV ring and form accessory electrical pathways, which could be located anywhere around the AV ring and may be multiple. During sinus rhythm, an atrial impulse will reach the ventricles via both the accessory pathway and the normal AV node.

These accessory pathways (APs) can conduct in both directions, from atria to ventricles (antegrade conduction) and from ventricles to atria (retrograde conduction). Approximately two thirds can only conduct retrogradely (concealed accessory pathways), and give rise to atrioventricular re-entrant tachycardias (AVRT), with normal EGCs in sinus rhythm. The remaining one third of APs are able to conduct antegradely during sinus rhythm producing ventricular pre-excitation. The ventricular complex on the ECG will be a fusion complex resulting from both conducting pathways, showing a shortened PR interval and a broad ventricular complex which form the characteristic so-called delta wave, which we call ventricular pre-excitation. When people with accessory pathways and antegrade conduction develop tachyarrhythmia, we call it the Wolff-Parkinson-White (WPW) syndrome (i.e. ventricular pre-excitation plus symptoms).

## Case Report

A 76-year-old man presented to the Emergency Department complaining of acute chest pain while he was dancing with his wife. He described his pain as a crushing precordial sensation with profuse sweating, without any radiation and not related to respiratory movements. It lasted for 30 minutes, being gradually relieved with rest. He denied palpitations or any other associated complaints.

He had a history of hypertension and epilepsy for which he was receiving treatment. No past history of ischemic heart disease was mentioned. He quit smoking many years ago and denied alcohol or drug consumption.

On admission in A&E, he was completely asymptomatic. On examination, he looked well, although he was tachycardic and haemodynamically compromised with a systolic blood pressure of 85 mmHg. The rest of his general clinical examination was unremarkable.

**Figure 1:**
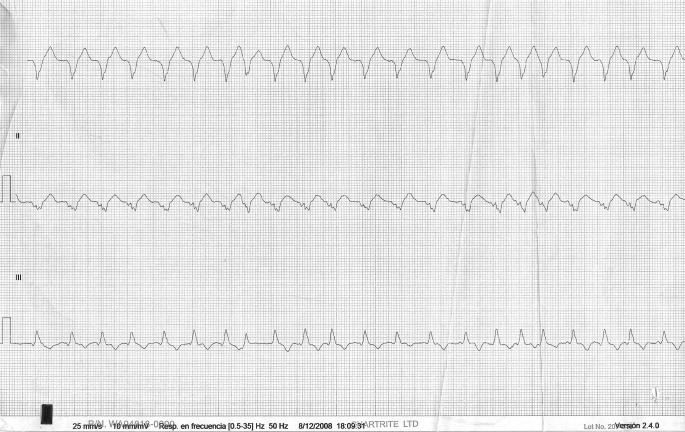
ECG in the Emergency Department, limb leads showing a broad complex tachycardia.

**Figure 2:**
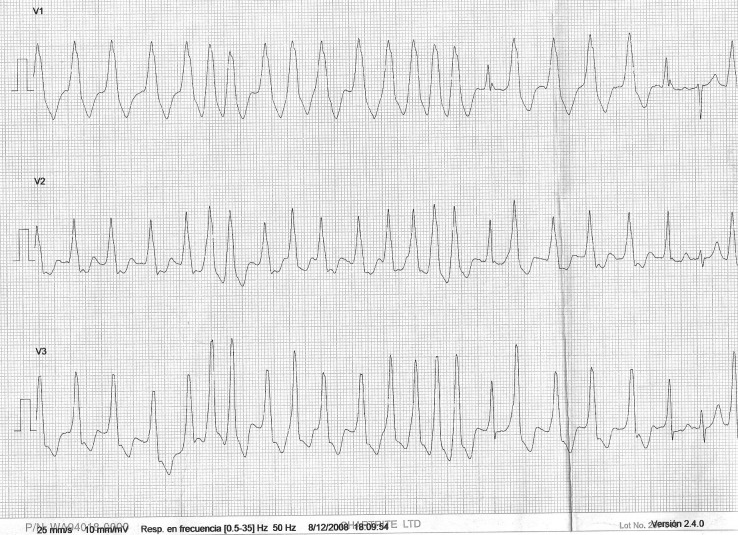
ECG in the Emergency Department, precordial leads showing pre-excited atrial fibrillation.

A 12-lead ECG showed a broad QRS complex tachycardia which at first sight resembled a ventricular tachycardia on limb leads (Fig. 1). On close inspection, the precordial leads displayed an irregularly irregular rhythm with broad and bizarre complexes at a rate of 145 (Fig. 2), alternating with a few narrow QRS complexes in sinus rhythm.

Blood glucose was elevated (without previous known diabetes mellitus) and the rest of blood tests were normal, including creatine kinase, cardiac troponin T, serum electrolytes and full blood count.

Given the patient risk profile (old age, hypertension, newly diagnosed diabetes), an acute coronary event was a concern and in the heat of the moment a decision for rapid reperfusion therapy was initially considered. It was then ruled out as the few normal complexes on the ECG showed no ST-segment elevation and an urgent transthoracic echocardiography demonstrated no regional wall abnormalities. The rest of the echocardiographic examination demonstrated mild concentric left ventricular hypertrophy with overall good systolic function and no significant valvular lesions.

He underwent DC cardioversion performed under intravenous sedation and reverted to sinus rhythm. The ECG post cardioversion demonstrated no pre-excitation.

**Figure 3:**
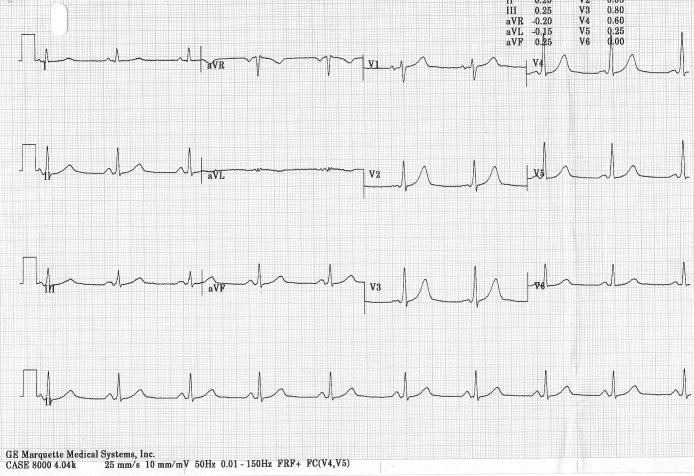
ECG two days after admission showing signs of ventricular pre-excitation.

He was admitted to CCU and continuous telemetry failed to demonstrate any significant cardiac arrhythmia during hospital stay. Cardiac troponin T raised at 0.42 µg/L after DC cardioversion and decreased to 0.17 µg/L in 8 hours, ruling out an acute coronary event again. Two days after admission the ECG showed signs of ventricular pre-excitation (Fig. 3), which were absent in previous recordings.

We contacted our regional reference Arrhythmia Unit and referred our patient for an electrophysiological study. A left lateral accessory pathway was located and successfully ablated.

## Discussion

Approximately 1.5-3 per 1000 of the population have the electrocardiographic signs of ventricular pre-excitation. One third of these will not develop arrhythmias, and will not need any treatment unless they work in high-risk occupations (pilots, school bus drivers, divers, sportspeople etc.), and two-thirds will experience cardiac arrhythmias, and so will be said to have the WPW syndrome. Patients with asymptomatic pre-excitation need no treatment and should be encouraged to seek medical expertise whenever arrhythmia related symptoms occur [1]. The treatment of choice for symptomatic patients with pre-excitation is destruction of the abnormal pathway by radiofrequency catheter ablation, which represents the cure for this condition [2].

With age, fibrosis may occasionally develop in the AV ring and block an accessory pathway. Thus, this congenital condition is more commonly first detected in young people, and it is rare that patients present with symptoms in older age as in our case.

Conduction via the accessory pathway may be small or big, depending on the amount of impulse conducted via the AP. Left-sided APs (like our patient’s) show lesser degrees of pre-excitation than right free-wall bypass tracts. Our patient also showed variable and intermittent degrees of pre-excitation. Ventricular pre-excitation may be an intermittent phenomenon. In the same ECG, therefore, we can see normal and pre-excited beats and some ECGs will show only normal beats or very small degrees of pre-excitation, as happened in our case where normal ECGs post cardioversion were recorded. Thus, physicians attending cases of tachycardia in acute settings must display a high grade of suspicion for this condition, closely inspecting post cardioversion and previous ECGs.

During atrial fibrillation (with up to 350-600 impulses/min) in patients without pre-excitation, the AV node protects the ventricle from the rapid atrial activity. In patients with WPW syndrome the accessory pathway provides an additional route of access to the ventricles and can conduct very rapidly in some patients, giving rise to very fast ventricular rates. Chaotic atrial activity can thus be fully transmitted to the ventricles causing a low output state and hypotension, or can degenerate in ventricular fibrillation and eventually lead to death. That is why this arrhythmia must be considered a life-threatening condition and physicians have to be fully aware of its existence and the subtleties of its management.

First of all, the rapid broad-complex tachycardia producing severe symptoms can be easily mistaken or misinterpreted as a ventricular tachycardia. The irregularity of RR intervals in pre-excited AF is the key difference with VT, which by definition has regular broad complexes. Again close inspection of ECG recordings must be undertaken, as the irregular pattern can simply be missed with very fast rhythms. In general, precordial leads will be more revealing for this purpose than limb leads as we show in this case. The bizarre appearance of the broad complexes in this tachycardia is caused by delta waves alternating with normal or near normal beats [3]. Once we have diagnosed an AF in a patient with WPW syndrome, the physician has to be aware of the differences in management with conventional non-pre-excited AF. AF in the setting of WPW tends to be worse tolerated than conventional AF, and the patient will more often be symptomatic or hypotensive, requiring us to perform sedation and synchronized DC cardioversion more frequently. If the patient is stable, the drugs of choice that block the accessory pathway are either IV procainamide or flecainide. IV Amiodarone can also be used. Drugs that are used as conventional treatment in AF, i.e. AV-blocking agents such as adenosine, calcium-channel blockers or digoxine, must be avoided at all costs. The rate of transmission through the accessory pathway likely would increase, having disastrous consequences in a person with AF in the setting of WPW, possibly causing the arrhythmia to deteriorate into ventricular fibrillation or refractory cardiac arrest. Notably, these AV blocking agents can be safely used in other more common arrhythmias in the WPW syndrome without pre-excitation, such as the narrow-complex orthodromic AV re-entrant tachycardia [4].

Very short R-R intervals with less than 250 msec correlate with poor prognosis and increased mortality, as is the case in our patient [5]. After being cardioverted to sinus rhythm, the patient should not be discharged home from A&E, as is standard practice for uncomplicated atrial fibrillations. He or she must be admitted to a unit with continuous telemetry and the reference arrhythmia team must be contacted for an electrophysiological study. Location and ablation of the accessory pathway should be undertaken during the current hospital admission in order to prevent recurrence of this dangerous arrhythmia in the discharged patient [6].
